# Reduced myocardial work in Fontan circulation: feasibility, reproducibility, and diagnostic performance

**DOI:** 10.1093/ehjimp/qyag073

**Published:** 2026-04-20

**Authors:** Jolanda Sabatino, Roberta Biffanti, Martina Avesani, Elena Reffo, Alessia Cerutti, Biagio Castaldi, Alice Pozza, Irene Cattapan, Elena Gribaudo, Valeria Pergola, Salvatore De Rosa, Daniele Torella, Giovanni Di Salvo

**Affiliations:** Department of Experimental and Clinical Medicine, Magna Graecia University, Viale Europa, 1,Catanzaro 88100, Italy; Cardiovascular Research Center, Magna Graecia University, Viale Europa, 1, Catanzaro 88100, Italy; Department for Women's and Children’s Health, University of Padua, Via Giustiniani, 2, Padua 35128, Italy; Pediatric and Congenital Cardiology Unit, University Hospital of Padua, Via Giustiniani, 2, Padua 35128, Italy; Pediatric Research Institute (IRP) ‘Città Della Speranza’, Corso Stati Uniti, 4F, Padua 35127, Italy; Pediatric and Congenital Cardiology Unit, University Hospital of Padua, Via Giustiniani, 2, Padua 35128, Italy; Department for Women's and Children’s Health, University of Padua, Via Giustiniani, 2, Padua 35128, Italy; Pediatric and Congenital Cardiology Unit, University Hospital of Padua, Via Giustiniani, 2, Padua 35128, Italy; Pediatric and Congenital Cardiology Unit, University Hospital of Padua, Via Giustiniani, 2, Padua 35128, Italy; Pediatric and Congenital Cardiology Unit, University Hospital of Padua, Via Giustiniani, 2, Padua 35128, Italy; Department for Women's and Children’s Health, University of Padua, Via Giustiniani, 2, Padua 35128, Italy; Pediatric and Congenital Cardiology Unit, University Hospital of Padua, Via Giustiniani, 2, Padua 35128, Italy; Department for Women's and Children’s Health, University of Padua, Via Giustiniani, 2, Padua 35128, Italy; Pediatric and Congenital Cardiology Unit, University Hospital of Padua, Via Giustiniani, 2, Padua 35128, Italy; Department for Women's and Children’s Health, University of Padua, Via Giustiniani, 2, Padua 35128, Italy; Pediatric and Congenital Cardiology Unit, University Hospital of Padua, Via Giustiniani, 2, Padua 35128, Italy; Pediatric Research Institute (IRP) ‘Città Della Speranza’, Corso Stati Uniti, 4F, Padua 35127, Italy; Pediatric Cardiology and Congenital Heart Disease Unit, Regina Margherita Children’s Hospital, Piazza Polonia, 94, Turin 10126, Italy; Department of Cardio-Thoraco-Vascular Sciences and Public Health, University of Padua, Via Giustiniani, 2, Padua 35128, Italy; Department of Experimental and Clinical Medicine, Magna Graecia University, Viale Europa, 1,Catanzaro 88100, Italy; Cardiovascular Research Center, Magna Graecia University, Viale Europa, 1, Catanzaro 88100, Italy; Department of Medical and Surgical Sciences, Magna Graecia University, Viale Europa, 1, Catanzaro 88100, Italy; Department of Experimental and Clinical Medicine, Magna Graecia University, Viale Europa, 1,Catanzaro 88100, Italy; Cardiovascular Research Center, Magna Graecia University, Viale Europa, 1, Catanzaro 88100, Italy; Department for Women's and Children’s Health, University of Padua, Via Giustiniani, 2, Padua 35128, Italy; Department for Women's and Children’s Health, University of Padua, Via Giustiniani, 2, Padua 35128, Italy; Pediatric and Congenital Cardiology Unit, University Hospital of Padua, Via Giustiniani, 2, Padua 35128, Italy; Pediatric Research Institute (IRP) ‘Città Della Speranza’, Corso Stati Uniti, 4F, Padua 35127, Italy

**Keywords:** Fontan, single ventricle, myocardial work, speckle tracking echocardiography, global longitudinal strain, ventricular function

## Abstract

**Aims:**

To evaluate the feasibility, reproducibility, and diagnostic performance of non-invasive myocardial work (MW) indices in patients with Fontan circulation compared with matched healthy controls.

**Methods and results:**

In this single-centre observational study, echocardiography and speckle-tracking analysis were performed in 70 Fontan patients (mean age 21 ± 9 years; 53% male) and age/sex/body size/blood pressure–matched controls. Global longitudinal strain (GLS) and MW indices—myocardial work index (MWI), constructive work (MCW), wasted work (MWW), and work efficiency (MWE)—were derived using vendor software and brachial cuff blood pressure. MW analysis was feasible in 86% of Fontan examinations. Compared with controls, Fontan patients had significantly lower MWI (1162 ± 364 vs. 1777 ± 240 mmHg%, *P* < 0.001), GLS (−13.9 ± 3.1% vs. −21.2 ± 1.5%, *P* < 0.001), and EF/FAC (58.9 ± 4.5% vs. 63.3 ± 3.9%, *P* = 0.002). MCW (1554 ± 450 vs. 2102 ± 221 mmHg%, *P* < 0.001) and MWE (90 ± 6% vs. 96 ± 2%, *P* < 0.001) were also reduced. MWI remained lower than controls even in Fontan patients with preserved EF/FAC. Inter-rater reliability was excellent for MWI (ICC 0.957; 95% CI 0.838–0.989) and good–excellent for GLS (ICC 0.898; 95% CI 0.641–0.974). Receiver operating characteristic analysis showed excellent discrimination for GLS (AUC 0.988) and MWI (AUC 0.925), and good discrimination for MCW (AUC 0.902) and MWE (AUC 0.795).

**Conclusion:**

Non-invasive MW indices are feasible and highly reproducible in Fontan circulation and identify reduced ventricular work and efficiency, including in patients with preserved conventional systolic measures. MW may provide a sensitive marker of subclinical myocardial dysfunction and support longitudinal follow-up in this population.

## Graphical Abstract

**Graphical Abstract:**
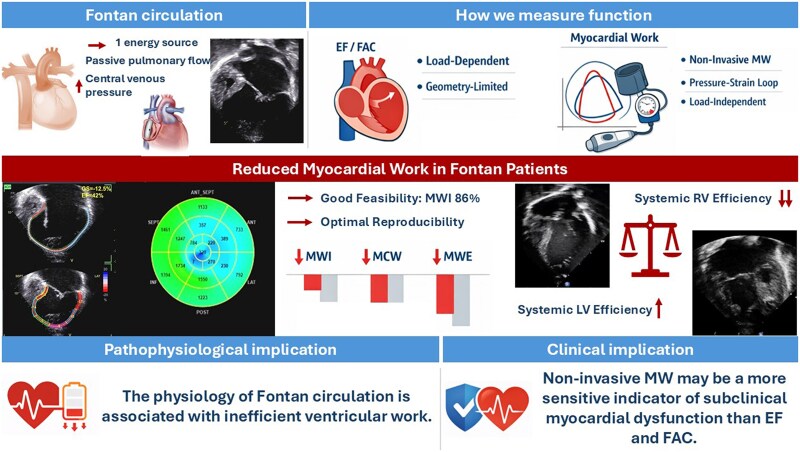
Visual summary of the study aims (assessment of non-invasive MW in Fontan circulation), key results (reduced MWI and MWE with good feasibility and reproducibility), and take-home message (MW may detect subclinical dysfunction beyond EF/FAC).

## Background

Children born with a functional single ventricle necessitate surgical palliation with the Fontan procedure, which is regarded as the standard of care for this population.^1–4^ The original Fontan operation, first performed in 1968 as an atrio-pulmonary connection, has since undergone numerous modifications, now comprising a total cavo-pulmonary connection, in which the systemic venous return from both the superior and inferior vena cava is directly routed to the pulmonary arteries. This configuration allows passive pulmonary blood flow driven by systemic venous pressure, thereby separating the systemic and pulmonary circulations in patients with single-ventricle physiology.^5^ As a consequence, Fontan physiology relies on chronically elevated central venous pressure, above normal physiological values, to sustain passive pulmonary blood flow and ensure adequate preload of the systemic ventricle.^5^

Although this procedure nearly normalizes arterial saturation and alleviates chronic cardiac volume overload, the likelihood of being free from any morbidity over time remains quite low. Common complications in these patients include cardiac failure, thromboembolism, arrhythmias, nephropathy, liver disease, and protein-losing enteropathy, as well as premature death and reduced exercise capacity.^6–8^ In addition to the haemodynamic abnormalities affecting the systemic venous circulation, patients who have undergone Fontan palliation are principally at risk for both systolic and diastolic dysfunction of the systemic ventricle.^9^

Assessing ventricular function in patients with functionally single ventricles^10^ and unusual ventricular geometry is challenging.^11,12^ Over the past decades, various techniques have been employed to estimate myocardial contractility and cardiac work.

Ventricular ejection fraction (EF) is the most commonly used surrogate marker of left ventricular function in many cardiac diseases due to its simple estimation and widespread use.^13^ However, EF calculation has several significant limitations: it is a volume-derived index, relies on geometric assumptions, can be challenging to calculate in patients with atypical ventricular anatomy, is extremely load-dependent, leading to considerable reproducibility loss, and does not reflect true LV contractility, making it poorly sensitive in detecting small changes in ventricular function.^13^

Myocardial work (MW) is defined as cardiac output multiplied by aortic pressure and is calculated as the area of the left ventricle pressure-volume curve measured during an invasive procedure.^14^ Although this parameter appears to be a sensitive predictor of myocardial work impairment,^15,16^ its invasive nature makes it unfeasible for routine assessments.

Recently, a novel non-invasive method for calculating MW has been introduced based on speckle- tracking analysis to estimate LV pressure from brachial artery cuff pressure.^16,17^ The strength of this method, unlike the ejection fraction, is its independence from loading conditions.^17^

To date, there have been no quantitative analyses on the effects of Fontan circulation on myocardial work.

Accordingly, the present study was designed to pursue the following objectives: (i) to assess the feasibility and reproducibility of non-invasive myocardial work indices; (ii) to compare MW between patients with univentricular hearts and healthy controls; and (iii) to evaluate the potential role of MW indices as markers of myocardial performance in this population.

## Methods

### Clinical data

Seventy consecutive patients who underwent a Fontan procedure between 1982 and 2017 at Padua Hospital were included in this cross-sectional study. Patients without available echocardiographic data were excluded, as were patients who had undergone a cardiac transplant or a redo-Fontan procedure.

All patients underwent elective hospitalization in the Paediatric Cardiology Unit between June 2021 and June 2022, during which the following examinations were collected:

✓ Clinical analysis (blood pressure, heart rate, resting oxygen saturation)
✓ Laboratory blood tests
✓ Transthoracic echocardiography

### Echocardiographic data

Echocardiographic examinations were performed by two expert cardiologists using the GE Vivid E9 ultrasound system (GE Healthcare, Chicago, IL). Both M5S-D and 6SD transducers (GE Healthcare) were used according to the patient's age and body size.

The following data were collected:

- Systemic atrioventricular valve (SAVV) regurgitation or stenosis (defined as absent, mild, moderate, or severe)
- Diastolic function, including E-wave velocity (cm/s), A-wave velocity (cm/s), E/A ratio, deceleration time (ms), E’-wave velocity (cm/s, measured at the free wall of the dominant ventricle), E/E’ ratio, D-wave deceleration time (ms)
- Diastolic function was also evaluated after the leg lifting test, with the legs being lifted passively by the clinician for one minute, to unmask latent diastolic dysfunction
- In patients with a left single ventricle, ventricular function was evaluated using MAPSE (mm) and ejection fraction (EF) with the Simpson method (%)
- In patients with a right single ventricle, the ventricular function was evaluated using TAPSE (mm) and fractional area change (FAC) (%)
- For the global longitudinal strain (GLS) and myocardial work evaluation, the apical chamber views were acquired with a frame rate of 60–100 frames/s. In some patients, due to complex cardiac anatomy or suboptimal echocardiographic windows, only a single view (usually apical four-chamber view) was analysed. The best-quality images were then transferred to an offline workstation (Echopac Version 202, GE Healthcare) to perform strain analysis using dedicated software.^17,18^ To calculate global and regional 2D STE myocardial longitudinal strain, the endocardial border was manually traced, and the region of interest automatically created by the software was then adjusted. Once strain analysis curves were obtained, a dedicated function of the GE software was used for MW estimation. The non-invasive blood pressure systolic and diastolic values, obtained by a brachial-cuff aneroid sphygmomanometer, were entered into the software. The time of opening and closure of the aortic and mitral valves was identified by the operator, as required for the synchronization of strain and pressure data. Global myocardial work index (MWI) was calculated as the area of the LV pressure-strain loops. From MWI, global constructive work (MCW), wasted work (MWW), and work efficiency (MWE) were estimated as:
- Global myocardial constructive work (MCW): work performed by a segment during shortening in systole plus negative work during lengthening in isovolumetric relaxation (IVR)
- Global myocardial wasted work (MWW): negative work performed by a segment during lengthening in systole plus work performed during shortening in IVR
- Global myocardial work efficiency (MWE): constructive work divided by the sum of constructive and wasted work (0–100%)

All the myocardial work data were compared with healthy age-, weight-, height-, blood pressure-, and sex-matched controls.

### Data analysis and statistics

Data distribution was assessed by visual inspection of frequency histograms. Continuous variables are presented as mean ± standard deviation (SD) for normal distribution or as median (interquartile range) for non-normal distribution. Categorical data are expressed as percentages. Comparisons between study groups were analysed using the unpaired *t*-test or the Mann–Whitney *U* test for normally and non-normally distributed continuous variables, respectively. Pearson’s correlation was used to assess correlations between quantitative variables, while the chi-square test (χ^2^) or Fisher's exact test was used for qualitative variables. The Kruskal–Wallis test was used to compare more than two groups.

Inter-rater reliability was assessed using the intraclass correlation coefficient (ICC). A two-way random-effects model was applied, assuming that both subjects and measurements were random factors, with an absolute agreement definition. Two independent observers re-analysed a random sample of 10 studies to estimate inter-rater reliability; average-measure ICCs refer to the mean of the two observers’ measurements.

All statistical analyses were performed using SPSS v.21 (SPSS Inc., Chicago, IL, USA). A two-tailed *P*-value of 0.05 was considered significant.

## Results

### Baseline demographic data

A total of seventy patients were included with an overall mean age of 21 ± 9 years (age range 10–47 years) (*Table 1*). Of these, 37 (53%) were males, and 33 (47%) were females. Patients under the age of 18 were 32 (46%), while those over the age of 18 were 38 (54%). The average time elapsed since the completion of Fontan palliation was 197 ± 103 months, with an interval between 57 and 480 months. Forty-nine (70%) patients received an extra-cardiac conduit, 15 (21%) a lateral intra-cardiac tunnel, and 6 (9%) patients received a Björk atrio-infundibular anastomosis. The mean age at admission was 21 ± 9 years.

**Table 1:** Demographic and clinical features of the study cohort

Demographics	Fontan population (*n* = 70)
Age (years) on hospital admission (mean ± SD)	21 ± 9
Age range (years)	10–47
Male (*n*, %)	37 (52.9%)
Years after surgery (mean ± SD)	16 ± 8.5
**Type of CHD (*n*, %)**	
TA	15 (21)
DORV	7 (10)
PA/IVS	8 (11)
HLHS	13 (19)
MA	4 (6)
DORV	3 (4)
CAVC unbalanced	5 (7)
DILV	11 (16)
Criss-cross heart	2 (3)
Other	2 (3)
**Systemic ventricle physiology (*n*, %)**	
SLV	38 (54)
SRV	25 (36)
Indeterminate	7 (10)
**Type of surgery (*n*, %)**	
TCPC, intracardiac conduit	15 (21)
TCPC, extracardiac conduit	49 (70)
Bjork’s atrio-infundibular anastomoses	6 (9)
**SpO2 (mean ± SD)**	92 ± 12.24
**NYHA class (*n*, %)**	
I	48 (72)
II-III	19 (28)
IV	0
**Biochemical data on admission** **(*n*, %); (mean ± SD)**	
NT-pro-BNP > 125 ng/L	37 (55)
Hb (male population, mg/dL)	19.2 ± 10.7
Hb (female population, mg/dL)	14.4 ± 1.63
**Treatment (*n*, %)**	
Warfarin	18 (26)
Cardioaspirin	42 (61)
Beta-blockers	9 (13)
Vasodilators (*n*; %)	22 (32)
**Echocardiographic data (mean ± SD)**	
EF in SLV (%)	58 ± 7
FAC in SRV (%)	40 ± 8
E/A (ratio)	1.6 ± 0.5
E/E’ right	10.7 ± 4.4
E/E’ left	8.4 ± 4.9
TAPSE in SRV (mm)	11.8 ± 2.9
MAPSE in SLV (mm)	13.9 ± 3.8

**Abbreviations:**  *n*, number; SD, standard deviation; CHD, congenital heart disease; TA, tricuspid atresia; DORV, double outlet right ventricle; PA/IVS, pulmonary atresia with intact ventricular septum; HLHS, hypoplastic left heart syndrome; MA, mitral atresia; CAVC, complete atrioventricular canal; DILV, double inlet left ventricle; SLV, systemic left ventricle; SRV, systemic right ventricle; TCPC, total cavopulmonary connection; SpO_2_, peripheral oxygen saturation; NYHA, New York Heart Association functional class; NT-proBNP, *n*-terminal pro–B-type natriuretic peptide; Hb, haemoglobin; EF, ejection fraction; FAC, fractional area change; E/A, early-to-late ventricular filling ratio; E/E’, ratio of early transmitral/transtricuspid flow velocity to early diastolic myocardial velocity; TAPSE, tricuspid annular plane systolic excursion; MAPSE, mitral annular plane systolic excursion.

Regarding the underlying cardiac anatomy, 13 (19%) patients were born with hypoplastic left heart syndrome, 11 (16%) with double-inlet left ventricle, 8 (11%) with pulmonary atresia with intact interventricular septum (PA/IVS), 15 (21%) with tricuspid atresia (TA), 5 (7%) with an unbalanced complete atrioventricular canal (CAVC-s), 3 (4%) with double outlet right ventricle (DORV), 4 (6%) with mitral atresia (MA), 2 (3%) with criss-cross heart, and 2 (3%) with other anomalies. A total of 25 (36%) patients have a single right ventricle (FSRV), 38 (54%) a single left ventricle (FSLV), and 7 (10%) an undetectable morphology (INDET).

Forty-eight (48/67, 72%) patients were in NYHA class I, while 19 (19/67, 28%) patients were in class II or III. In three patients, it was not possible to estimate the functional NYHA class due to severe neuro-psychomotor impairment. NT pro-BNP was measured in 66 patients, and it turned out to be elevated (>125 ng/L) in 37 (55%): 17 with a functional single left ventricle and 20 with a functional single right ventricle.

### Echocardiographic data

For patients with a functional left single ventricle, the mean EF was 58 ± 7%, with only 3 (8%) patients having an EF below 50%. For those with a right single ventricle, the mean FAC was 40 ± 8% with 7 (23%) patients having a pathological FAC (below 35%).

Eleven patients (11/68, 16%) had a normally functioning SAVV, 50 patients (74%) had mild SAVV regurgitation, 6 (9%) had moderate SAVV regurgitation, and 1 (1%) had severe SAVV regurgitation. All patients with a functional left single ventricle had no more than mild SAVV regurgitation, while five patients (20%) of those with a functional right single ventricle had more than moderate SAVV regurgitation.

Regarding diastolic function, it was evaluated in 63 patients (90%). The E/A ratio was pathological (E/A < 1) in four patients (6%), while the E/E’ ratio was pathological (E/E’>12) in 11 patients (17%). However, if the diastolic function is evaluated according to Margossian's classification for Fontan patients,^19^ only 34 (54%) patients had normal diastolic function.

The leg lifting test was performed on 45 patients (64%). After performing the test, the E/E’ ratio increased in 22 (49%) patients, and, according to Margossian’s classification,^19^ only 16 patients (35%) maintained normal diastolic function. A strong correlation was detected between the E/E’ ratio before and after the leg lifting test (Pearson's *r* 0.847, *P* < 0.01).

### Global longitudinal strain and global myocardial work parameters

**Figure 1:**
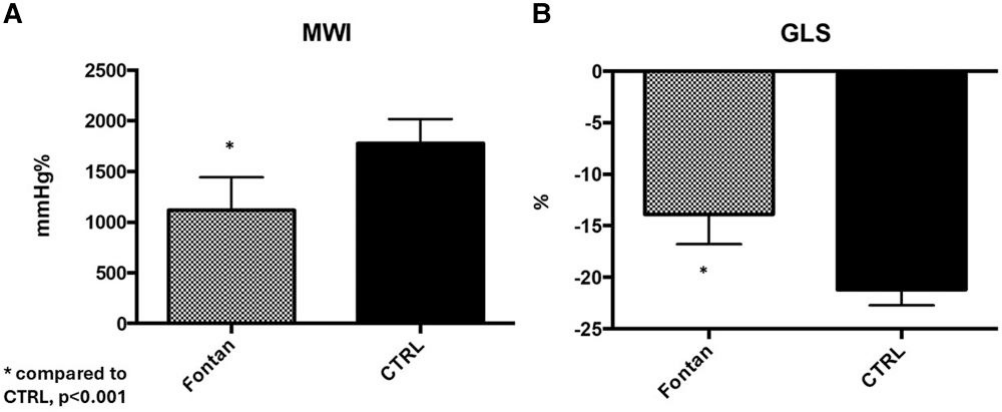
Comparison of MWI and GLS between Fontan patients and matched healthy controls (CTRL). Panel A: MWI (mmHg%). Panel B: GLS (%). Values are mean ± SD. *P < 0.001 vs. CTRL.

**Figure 2:**
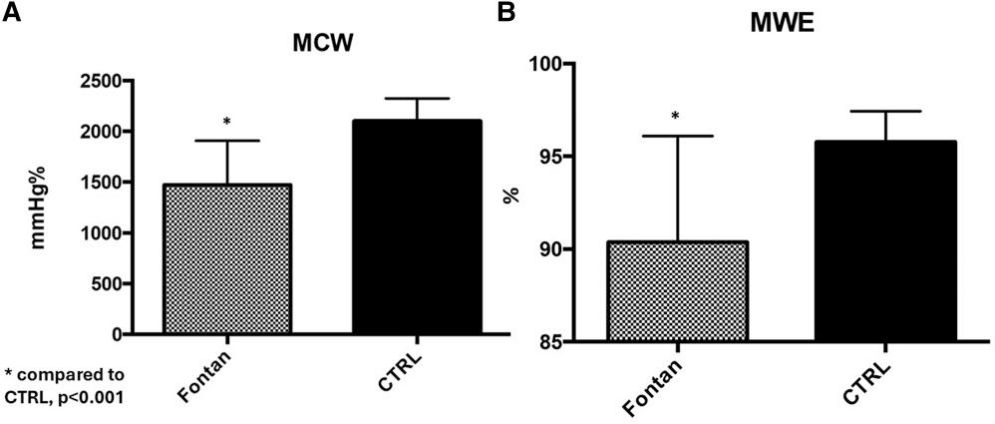
Comparison of MCW and MWE between Fontan patients and matched healthy controls (CTRL). Panel A: MCW (mmHg%). Panel B: MWE (%). Values are mean ± SD. *P < 0.001 vs. CTRL.

The mean MWI in Fontan patients was 1162 ± 364 mmHg%, significantly lower (*P* < 0.001) compared to the control healthy population (CTRL) (1777 ± 240 mmHg%) (*Figure 1*). The mean GLS (−13.9 ± 3.1% vs. −21.2 ± 1.5%, *P* < 0.001) and the EF (58.9 ± 4.5% vs. 63.3 ± 3.9%, *P* < 0.002) were significantly lower in Fontan patients compared with healthy CTRL (*Figure 1*). In Fontan patients, the mean MCW was 1554 ± 450 mmHg%, significantly reduced compared to the CTRL (2102 ± 221 mmHg%, *P* < 0.001). Also, the MWE was significantly lower (*P* < 0.001) in the Fontan population (90 ± 6%) than in the CTRL (96 ± 2%) (*Figure 2*).

**Figure 3:**
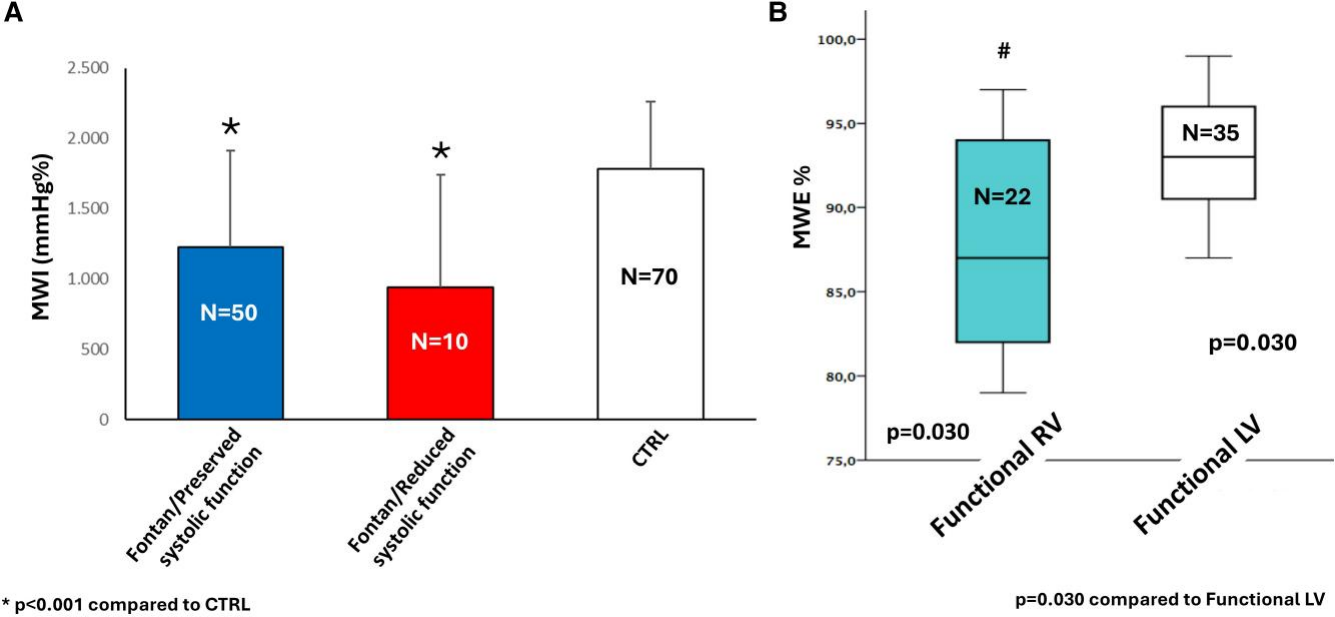
Subgroup analyses of myocardial work parameters in Fontan patients [MW was feasible in *n* = 60 (86%) Fontan patients]. Panel A: MWI across Fontan patients with preserved EF/FAC, Fontan patients with reduced EF/FAC, and controls (CTRL). Panel B: MWE according to systemic ventricular morphology (functional single left ventricle vs. functional single right ventricle). Exact *P*-values are shown on the figure; *P < 0.001 vs. CTRL; *P* = 0.030 between ventricular morphology subgroups.

In Fontan patients with preserved ejection fraction or FAC (LVEF >50%, FAC >35%), MWI values were significantly lower compared with controls (*P* < 0.001) (*Figure 3*).

Moreover, Fontan patients with a functional right ventricle showed significantly reduced MWE compared with those with a functional left ventricle (*P* = 0.030) (*Figure 3*). Moreover, in the subgroup of patients with a functional right ventricle, an increased E/E’ was observed (*P* < 0.001). No other significant differences were observed between the two subgroups in terms of MWI, MCW, MWW, and E/A.

A moderate correlation between MWI and FAC (Pearson's *r* 0.535, *P* = 0.027) and between MCW and MAPSE (Pearson's *r* 0.450, *P* = 0.036) was observed.

### ROC analysis for discriminating Fontan patients

**Figure 4:**
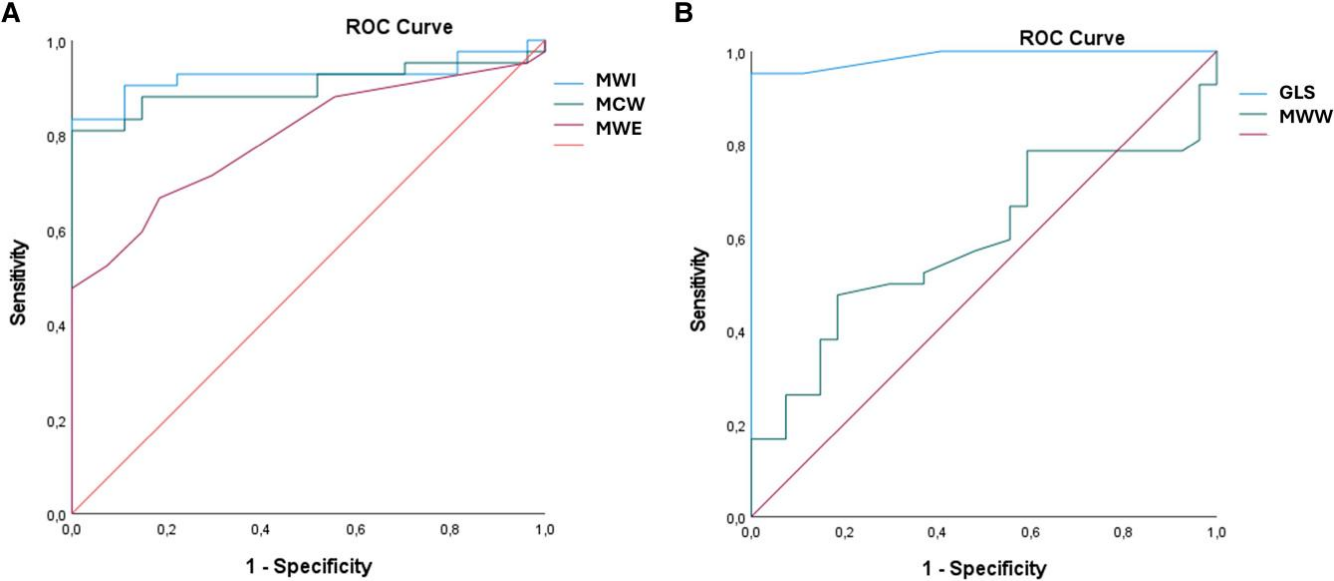
Receiver operating characteristic (ROC) curves evaluating the ability of GLS and MW-derived indices to discriminate Fontan patients from controls. AUC values: GLS 0.988, MWI 0.925, MCW 0.902, MWE 0.795, and MWW 0.592.

Receiver operating characteristic (ROC) analysis was performed to evaluate the ability of GLS and MW–derived indices to discriminate patients with univentricular hearts (*Figure 4*). The analysis demonstrated excellent diagnostic performance for GLS and MWI (AUC = 0.988 and 0.925, respectively), good discriminatory ability for MCW and MWE (AUC = 0.902 and 0.795, respectively), and only modest performance for MWW (AUC = 0.592).

### Reproducibility and feasibility

The evaluation of MWI and GLS was feasible in 86% and 86% of patients with morphologic LV, and in 84% and 84% of patients with morphologic RV, respectively. The feasibility of MW analysis was high and comparable to that of GLS. The main reasons for non-feasibility included suboptimal acoustic windows and inadequate speckle-tracking quality, especially in patients with an obese and/or enphysematous body habitus and/or prior surgical complexity. Notably, MW analysis was feasible in all patients in whom GLS measurement could be successfully obtained, and no specific patient characteristics were consistently associated with non-feasibility. There was no significant difference in the feasibility of assessment of GLS and MW indices based on ventricular morphology (*P* < 0.05).

For single measurements of the MWI, the ICC was 0.957, with a 95% confidence interval of [0.838–0.989], indicating excellent reliability. For average measurements, the ICC was 0.978 [95% CI (0.912–0.995)]. The *F* test against a true value of zero was statistically significant, *F* (9, 9) = 41.41, *P* < 0.001, confirming that agreement for the MWI was significantly greater than chance.

The ICC for GLS was 0.898 for single measures [95% CI (0.641–0.974)] and 0.946 for average measures [95% CI (0.782–0.987)], *F*(9, 9) = 16.96, *P* < 0.001.

## Discussion

In this study, we have assessed the feasibility, reproducibility, and diagnostic value of non-invasive MW indices in patients with Fontan palliation. The main findings are as follows: (i) the assessment of non-invasive MW indices was feasible in 85% of the patients, had good reproducibility within and between observers, and had evaluate the ability to discriminate patients with univentricular hearts with optimal accuracy for MWI and MCW (AUC = 0.925 and 0.902, respectively); (ii) the physiology of Fontan circulation is associated with inefficient ventricular work. MWI values were lower in Fontan patients compared to healthy controls, even in those with preserved EF or FAC, suggesting that non-invasive MW indices may be a more sensitive indicator of subclinical myocardial dysfunction than conventional parameters; and (iii) the patients with morphologic RV had a higher prevalence of impaired MWE compared to those with morphologic LV (*P* = 0.030).

Although the haemodynamic disturbances characteristic of Fontan physiology may primarily involve the systemic venous circulation, a subset of patients also develops systolic dysfunction of the systemic ventricle. This ventricular impairment has been attributed to multiple factors^20–22^ including (i) prior myocardial injury related to chronic ventricular volume overload and/or hypoxic damage secondary to cyanosis; (ii) ischaemic injury sustained during cardiopulmonary bypass in previous surgical procedures; (iii) chronic volume overload due to atrioventricular valve regurgitation, particularly common among individuals with a morphologic systemic right ventricle; and (iv) the high prevalence of rhythm disturbances, such as atrial arrhythmias and the need for ventricular pacing, which may precipitate or worsen ventricular dysfunction.

Patients undergoing Fontan palliation typically present with highly complex forms of congenital heart disease, resulting in substantial variability in ventricular morphology. Consequently, accurate evaluation of ventricular systolic function remains particularly difficult in this population, as it is unclear whether the geometric assumptions that underlie echocardiography-derived indices of systolic performance apply to such anatomically diverse ventricles.

Li and colleagues^23^ evaluated systemic ventricular global longitudinal strain in a cohort of 28 patients who had undergone Fontan palliation and compared the findings with those from 26 healthy control subjects. The Fontan group exhibited significantly lower absolute values of global longitudinal strain than the control group, indicating impaired systolic function. In a study of 512 patients by Aboelmaaty and colleagues,^24^ echocardiographic indices such as longitudinal strain could be obtained in more than 87% of participants, demonstrated good reproducibility, and showed modest correlations with cardiac magnetic resonance–derived ejection fraction, with threshold values of Echo-LS <15% provided the optimal discrimination for identifying patients at highest risk of death or transplantation.

The myocardial work index, as a method for evaluating cardiac performance, has demonstrated good reproducibility in previous works in paediatric patients.^17,18^ Moreover, as a non-invasive approach, it provides a straightforward analysis of myocardial function and workload in paediatric populations.^17,18^ MWI may be a more sensitive indicator of subclinical myocardial dysfunction than ejection fraction EF.^25,26^ Even with a normal EF, MWI values were lower in Fontan patients compared to healthy controls. This suggests that the conventional EF, calculated using Simpson’s method or conventional FAC, may not adequately reflect myocardial work in Fontan patients, as the geometry of the single ventricle does not align with the mathematical assumptions underlying EF calculation.

In this study, only a small proportion of patients exhibited compromised ventricular function when assessed using ejection fraction or fractional area change. However, when more sensitive parameters such as global longitudinal strain or myocardial work index were employed, a significant impairment in myocardial function was detected in a large proportion of patients, including those with normal EF or FAC values. Finally, ROC analysis primarily demonstrates diagnostic performance and construct validity of MW indices in detecting abnormal ventricular mechanics. The morphologic and demographic profile of the Fontan population has evolved considerably over time, reflecting the shift from atriopulmonary Fontan connections to total cavopulmonary connections, as well as a progressive increase in the proportion of patients with a morphologic right ventricle, largely attributable to the widespread adoption of the Norwood procedure. These demographic changes carry important clinical implications, as the presence of a morphologic systemic right ventricle has been consistently associated with a greater burden of systolic dysfunction and less favourable clinical outcomes. This is likely due to the anatomy of the right ventricle, which is less capable of supporting systemic circulation as effectively as the left ventricle. Aboelmaaty *et al*.^24^ found that patients with a morphologic right ventricle exhibited lower echocardiography-derived indices of systolic performance and, consequently, a higher prevalence of ventricular systolic dysfunction compared with those with a morphologic left ventricle.

In line with previous reports, our findings indicate that patients with a right systemic ventricle exhibit less favourable myocardial work performance, with significantly lower MWE, compared with patients with a left systemic ventricle.

This finding indicates that echocardiography-derived indices of MW represent reliable tools for the evaluation and longitudinal monitoring of ventricular systolic performance in the growing population of Fontan patients, both with a morphologic systemic right and left ventricle. Given the increasing prevalence of the both ventricular phenotypes in contemporary Fontan cohorts, the applicability of these measures across different ventricular morphologies has important clinical relevance and supports their use in routine follow-up and risk stratification.

We acknowledge that, as a cross-sectional study, our findings do not provide longitudinal outcome data and therefore cannot establish prognostic implications. Nevertheless, the study offers important insights into the feasibility and reproducibility of MW assessment in the Fontan population, a context in which comprehensive, multiparametric evaluation is particularly critical. MW indices should thus be regarded as sensitive functional markers capable of detecting subclinical ventricular dysfunction beyond conventional echocardiographic parameters. While these results highlight the potential utility of MW for detailed functional characterization, integration into clinical decision-making, such as guiding surveillance, timing of intervention, or risk stratification, will require confirmation through prospective, longitudinal studies designed to evaluate associations with relevant clinical outcomes, including ventricular failure, arrhythmias, and Fontan-related complications.

## Limitations

The principal limitations of this single-centre study include the relatively small sample size, which may limit statistical power and reduce the generalizability of the findings, and suboptimal echocardiographic image quality resulting from prior cardiac surgeries. Surgical scarring and altered thoracic anatomy frequently led to poor acoustic windows, potentially affecting the feasibility and accuracy of echocardiography-derived measurements and introducing measurement variability.

Moreover, the heterogeneity of the Fontan population, including differences in underlying cardiac anatomy, systemic ventricular morphology, and surgical technique, represents an important factor influencing MW measurements. Variations in ventricular geometry and loading conditions may affect MW indices and their interpretation, highlighting the need for careful contextualization when assessing ventricular function in this population. Although subgroup analyses by ventricular morphology were performed, the limited sample size restricts definitive conclusions. These considerations suggest that morphology-specific reference ranges or cut-off values may be necessary and should be explored in future multicentre studies to optimize the clinical utility of MW assessment in Fontan patients.

Another limitation is related to a methodological aspect to consider: MW estimation relies on brachial cuff systolic blood pressure as a non-invasive surrogate of ventricular pressure. While this approach is widely used in non-invasive MW assessment, its application in Fontan physiology may be influenced by the unique haemodynamic characteristics of this circulation, including the absence of a subpulmonary ventricle and altered loading conditions. In this context, the Fontan population may represent an ideal setting to further investigate the relationship between non-invasively derived MW indices and directly measured ventricular pressures. Future studies integrating echocardiographic MW analysis with invasive haemodynamic assessment could therefore provide valuable validation and help refine the interpretation of MW parameters in this complex physiology.

Finally, the ROC analysis performed against healthy controls demonstrates the diagnostic performance and construct validity of MW indices in identifying abnormal ventricular mechanics. However, we recognize that it provides limited information regarding their clinical utility within the Fontan population itself. The more clinically relevant question is whether MW parameters can stratify risk or predict adverse outcomes among Fontan patients. Addressing this issue will require prospective, longitudinal studies specifically designed to evaluate associations between MW indices and outcome-driven clinical endpoints.

## Conclusion

Fontan physiology is associated with reduced and inefficient ventricular work, even when conventional systolic indices (EF or FAC) remain within the normal range. Non-invasive myocardial work analysis was feasible in most Fontan studies and showed excellent inter-rater reproducibility. Myocardial work index, constructive work, and work efficiency discriminated Fontan patients from healthy controls, with the lowest efficiency observed in patients with a systemic right ventricle. These findings support echocardiography-derived myocardial work as a sensitive marker of subclinical myocardial dysfunction and a potential tool for risk stratification and longitudinal follow-up in Fontan circulation, thereby potentially allowing for earlier initiation of targeted therapy or optimization of heart transplant timing.

## Lead author biography


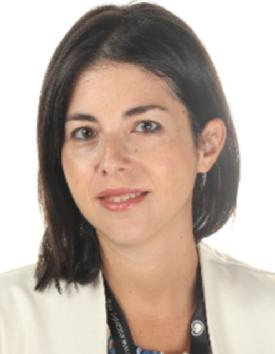
Dr. Jolanda Sabatino is a Cardiologist and Associate Professor at the University Hospital of Catanzaro, Italy. She earned her medical degree and Ph.D. in biomarkers of chronic and complex diseases from the Università degli Studi ‘Magna Græcia’ di Catanzaro, with board certification in cardiovascular diseases in 2017.

Her academic path spans internationally, including research fellowships at the Royal Brompton Hospital of London. She later joined the University of Padua’s programme in Paediatric Cardiology and Adult Congenital Heart Disease as an Assistant Professor.

Dr. Sabatino’s expertise lies in non-invasive cardiovascular imaging, advanced echocardiography in congenital heart disease, biomarkers, cardiomyopathies, and valve disease. She has authored over 100 publications and earned multiple accolades, including the Italian Society of Cardiology’s Young Investigator Award, an ESC Best Poster Award, and an ESC Training Grant in 2019.

Languages: Italian and English. Hobby: tennis

## Data Availability

The data underlying this article will be shared on reasonable request to the corresponding author.
